# Evaluate the guide RNA effectiveness *via Agrobacterium*-mediated transient assays in *Nicotiana benthamiana*

**DOI:** 10.3389/fpls.2023.1111683

**Published:** 2023-02-20

**Authors:** Zhibo Wang, Zachary Shea, Qi Li, Kunru Wang, Kerri Mills, Bo Zhang, Bingyu Zhao

**Affiliations:** School of Plant and Environmental Sciences, Virginia Tech, Blacksburg, VA, United States

**Keywords:** CRISPR/Cas9, guide RNA (gRNA), *Agrobacterium*-mediated transient assay, *N. benthamiana*, soybean

## Abstract

CRISPR/Cas9-based genome editing system is a powerful tool for plant genetic improvement. However, the variable efficiency of guide RNA(s) (gRNA) represents a key limiting factor that hampers the broad application of the CRISPR/Cas9 system in crop improvement. Here, we employed the *Agrobacterium*-mediated transient assays to evaluate the effectiveness of gRNAs for editing genes in *Nicotiana benthamiana* and soybean. We designed a facile screening system based on indels that can be introduced by CRISPR/Cas9-mediated gene editing. A gRNA binding sequence (23 nucleotides) was inserted into the open reading frame of yellow fluorescent protein (YFP) gene (gRNA-YFP), which disrupted the YFP reading frame and results in no fluorescent signal when it was expressed in plant cells. Transiently co-expression of Cas9 and a gRNA targeting the gRNA-YFP gene in plant cells could restore the YFP reading frame and recover the YFP signals. We evaluated five gRNAs targeting *Nicotiana benthamiana* and soybean genes and confirmed the reliability of the gRNA screening system. The effective gRNAs targeting *NbEDS1, NbWRKY70*, *GmKTI1*, and *GmKTI3* had been used to generate transgenic plants and resulted in expected mutations on each gene. While a gRNA targeting *NbNDR1* was confirmed to be ineffective in transient assays. This gRNA indeed failed to trigger target gene mutations in stable transgenic plants. Thus, this new transient assay system can be used to validate the effectiveness of gRNAs before generating stable transgenic plants.

## Introduction

The Clustered Regularly Interspaced Short Palindromic Repeat (CRISPR) Cas system has emerged as a powerful tool for gene editing in different organisms (Liu and Fan, 2014; Chen et al., 2019). It has been applied to improve the agronomic traits of many crop plant species (Chen et al., 2019; Wang et al., 2022 (under review)). New gene-editing tools, including base editing and prime editing, are continuously developed in both academic and private sectors (Zong et al., 2022).

CRISPR/Cas9 machinery employs short guiding RNA (gRNA) that has an 18-22 nucleotide (nt) region complementary to a short fragment in the gene of interest (Wu et al., 2014; Matson et al., 2019). With the navigation by gRNA, endonuclease Cas9 or its orthologues can bind and cleave target DNAs and introduce mutations at a desired location (Abudayyeh et al., 2016; Kim et al., 2017). The success of CRISPR/Cas9-mediated gene editing depends on recognizing the target DNA controlled by RNA-DNA interaction in the CRISPR/Cas9 system. Although the choice of gRNA would seem to be unlimited, studies in various organisms indicate that selection of gRNA significantly affects the efficiency of CRISPR/Cas9 targeting and/or DNA cleavage at target loci (Doench et al., 2014; Liang et al., 2016). Recent studies suggest that the nucleotide composition, length, and secondary structures of gRNAs can impact the specificity and efficiency of gene editing (Dang et al., 2015). Therefore, selection of an effective gRNA is critical for a successful gene editing experiment. In fact, the effectiveness of a gRNA is pivotal for the prime editing systems, which currently have low gene-editing efficiency (Chen et al., 2021a). For some plant species, such as apple, pepper, soybean, etc., the development of transgenics can take years to obtain stable transgenic plants. Therefore, selection of functionally validated Cas9/gRNA transformation constructs before the initiation of plant transformation process, will significantly increase the productivity of CRISPR/Cas9-mediated gene editing in crop plants.

The computational and experimental attempts have both been documented to evaluate the effectiveness of gRNAs in CRISPR/Cas9-mediated genome editing (Liang et al., 2016; Liu et al., 2020a; Xiang et al., 2021; Konstantakos et al., 2022). For example, Liang et al. (2016) proposed that the G/C nucleotide content of gRNA sequences should be around 30%-80%. The potential secondary structures in gRNAs may also interfere with their target recognition (Liang et al., 2016). Konstantakos et al. (2022) summarized the currently known computational tools that can be used to select effective gRNAs. Thus far, only two methods have been developed for the experimental evaluation of gRNAs in plant cells.The first one is utilizing a protoplast-based transient assay(Carlsen et al., 2021). However, these kind of protocols need DNA amplification and enzyme digestion and/or sequencing assistance to validate the effectiveness of gRNAs, which is inconvenient and cost-prohibitive (Liang et al., 2016; Liu et al., 2020a). The second methodis based on small insertions and deletions (indels) that can be introduced by CRISPR/Cas9-mediated gene editing, which is also the principle we employed in this study. In brief, Liang et al. (2019) developed a reporter construct that carries the gene of interest (GOI) fused with a site-specific endoribonuclease Csy4. A green fluorescent protein gene (GFP) that carries the Cys4 recognition site was cloned adjacent to the GOI-Csy4 Fusion gene. When there is no gene editing event, the GFP transcripts will be destroyed by Csy4. When a gene-editing event occurs on the GOI, small indels generated by CRISPR/Cas9 system have a 2/3 chance of stopping the expression of Csy4, therefore, preventing the degradation of*GFP* transcripts and resulting in increased GFP signals(Liang et al., 2019). However, this sophisticated system is not straightforward, which can be further simplified for high-throughput screening assays.

Small insertions and deletions can be introduced by the nonhomologous end-joining (NHEJ) during the process of CRISPR/Cas9-mediated gene editing (Kumar et al., 2019). Some INDELS cause a frameshift of the targeting gene at the gRNA binding site (Kumar et al., 2019), resulting in the loss of function of a target gene. On the other hand, certain INDELS can also correct the pre-existed frameshift mutation of a targeting gene, therefore, restore the gene functionality. Based on this mechanism, we developed a more convenient protocol to evaluate the effectiveness of gRNAs *via Agrobacterium*-mediated transient assays in *Nicotiana benthamiana*. The newly developed system can aid in the assessment of gRNA efficiency before the initiation of stable transformation. We validated the functionality and reliability of the screening system by testing gRNAs targeting three genes from *N. benthamiana* and two genes from soybean. This new system does not require sequencing of a target gene, and it is expected to be broadly applicable for gRNA validation in genome editing experiments.

## Results and discussions

### Two gRNAs targeting NbEDS1 and NbNDR1, respectively, have different effectiveness in terms of triggering the Cas9-mediated gene editing

The enhanced disease susceptibility 1 (*EDS1*) and nonrace-specific disease resistance 1 (*NDR1*) genes encode two key components of plant immunity, respectively, which are required for plant disease resistance in diverse plant species (Aarts et al., 1998; Coll et al., 2011; Knepper et al., 2011; Lapin et al., 2020). In a previous research effort, we aimed to develop *N. benthamiana* mutant plant that is impaired in both *NbEDS1* and *NbNDR1* genes. We manually designed two gRNAs targeting *NbEDS1* and *NbNDR1*, respectively, based on the location of the PAM sequence (NGG). They were cloned as a tandem array in one CRISPR/Cas9 construct (pCas9-*NbEDS1*/*NbNDR1* gRNA) and transformed into *N. benthamiana* plant (**Figure S1** and **Figure S2A**). In this construct, a U6 promoter is assigned to express each gRNA (**Figure S2A**), thus expressions of two gRNAs are ensured for the editing on both *NbEDS1* and *NbNDR1* genes. More than twenty T1 transgenic plants were recovered and genotyped. The *NbEDS1* genes were successfully edited in all genotyped transgenic plants (**Figure S2B**), while no edited *NbNDR1* genes were detected in any genotyped transgenic plants, even in the T2 and T3 generations (**Figure S2C**). Interestingly, we noticed that the *NbNDR1* gRNA sequence has 70% AT; the low GC content may suggest it has a low binding affinity to the target gene (Liang et al., 2016; Graf et al., 2019). We speculated that the gRNA targeting *NbEDS1* is more effective than the one targeting *NbNDR1*.

Previous reports suggest that the mutation of *NbEDS1* can suppress *N. benthamiana* disease resistance to *Xanthomonas euvesicatoria* (*Xe*) (Schultink et al., 2017; Martin et al., 2020). We inoculated the pCas9-*NbEDS1*/*NbNDR1* gRNA transgenic plants with *Xe* strain *Xe85-10*. All tested transgenic plants, but not the wild-type plants, were susceptible to the infection of *Xe85-10*, resulting in an apparent water-soaking phenotype at the inoculation sites (**Figure S2D**). *NbNDR1* is required for the Rps2 (resistance to *P*. *syringae*)/AvrRpt2-mediated disease resistance (Century et al., 1995; Aarts et al., 1998; Knepper et al., 2011; Chen et al., 2021b). Therefore, we also inoculated pCas9-*NbEDS1*/*NbNDR1* gRNA transgenic plants, along with wild-type controls, with *Agrobacterium tumefaciens* strains carrying the 35S::*RPS2* and 35S::*avrRpt2*. As expected, transient co-expression of *Rps2* and *avrRpt2* genes triggered a programmed cell death phenotype on both transgenic and wild-type plants (**Figure S2E**). This result suggests that *NbNDR1* was not mutated, which is consistent with the DNA sequencing result (**Figure S2C**). Thus, our data suggest that even though two gRNAs targeting *NbEDS1* and *NbNDR1*, respectively, were assembled into one transformation construct, the effectiveness of two gRNA-triggered gene editing is different, where only *NbEDS1-*gRNA triggers the gene editing events.

**Figure 1:**
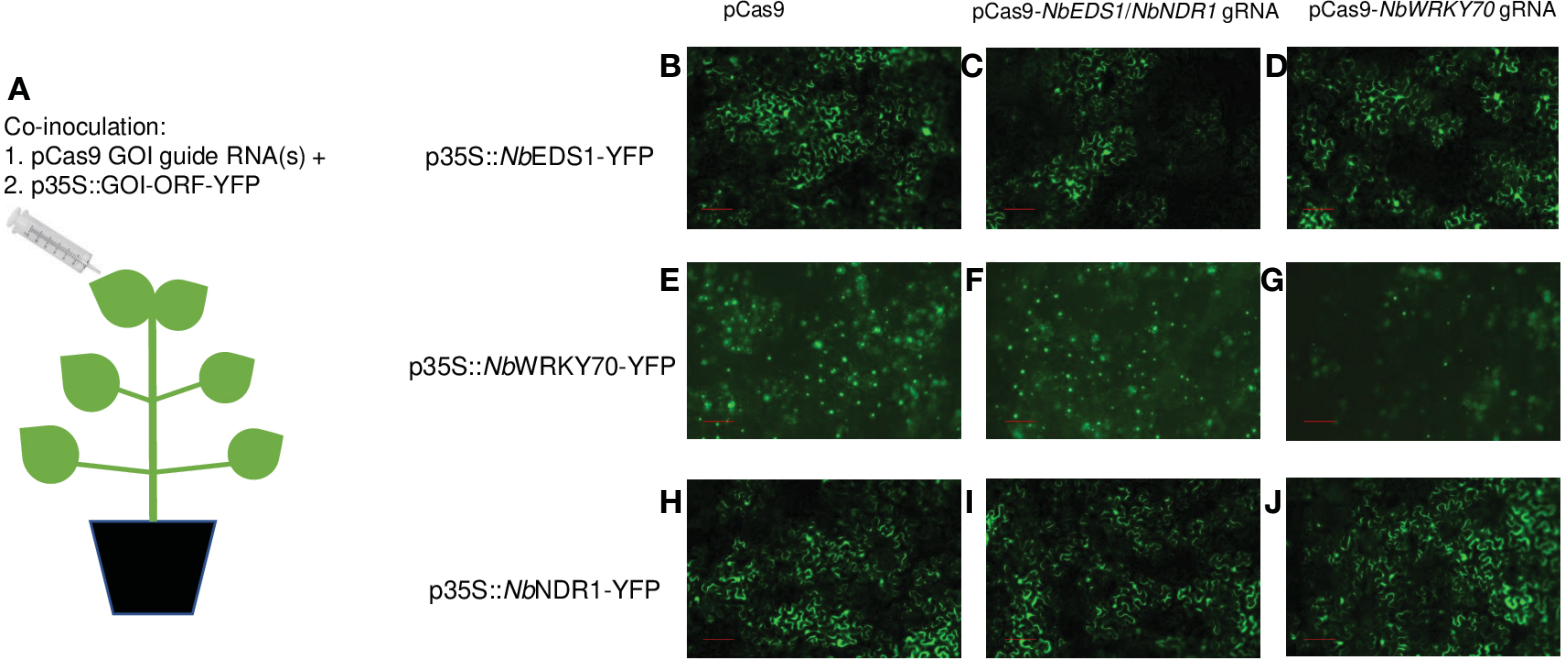
Evaluate the effectiveness of gRNAs targeting *NbEDS1*, *NbNDR1*, *NbWRKY70 via Agrobacterium*-mediated transient assay. **(A)** The co-inoculation scheme of *A. tumefaciens* strains carrying p*Cas9*-*GOI* gRNA and p35S::*GOI-YFP* on *N. benthamiana*. p35S::*NbEDS1-YFP* **(B–D)**, p35S::*NbWRKY70-YFP* **(E–G)**, and p35S::*NbNDR1-YFP* **(H–J)** were respectively co-inoculated with pCas9 empty vector (no gRNA), p*Cas9*-*NbEDS1/NbNDR1* gRNAs, and p*Cas9*-*NbWRKY70* gRNA. The YFP signals were detected by a fluorescent microscope at 48 hour post inoculation. *NbEDS1* gRNA and *NbWRKY70* gRNA, but not *NbNDR1* gRNA, can trigger gene editing when co-expressing with pCas9-GOI gRNA in *Agrobacterium*-mediated transient assays in *N. benthamiana*, which resulted in reduced fluorescent signals. Scale bar represents 100 µm. GOI; gene of interest, YFP; yellow fluorescent protein, 35S; Cauliflower mosaic virus 35S promoter.

To clarify our speculation, we performed *Agrobacterium*-mediated transient assays on *N. benthamiana*, and the co-inoculation diagram can be visualized in **Figure 1A**. *A. tumefaciens* strains carrying p35S::*NbEDS1*-yellow fluorescent protein (YFP) or p35S::*NbNDR1*-*YFP* with or without pCas9-*NbEDS1*/*NbNDR1* gRNA, were inoculated onto the *N. benthamiana* leaf. We previously also generated transgenic *N. benthamiana* plants expressing pCas9-*NbWRKY70* gRNA, where the *NbWRKY70* gene was successfully knocked out, suggesting that *NbWRKY70* gRNA is functional and efficient (Liu et al., 2016). Therefore, in this study, we also used pCas9-*NbWRKY70* gRNA and the p35S::*NbWRKY70*-YFP as controls.

The fluorescent signals of *Nb*EDS1-YFP, *Nb*NDR1-YFP, and *Nb*WRKY70-YFP fusion proteins were detected under a fluorescent microscope (**Figures 1B-J**). The fluorescent signals of *Nb*EDS1-YFP slightly reduced when it was co-expressed with pCas9-*NbEDS1*/*NbNDR1* gRNA, but not pCas9 or pCas9-*NbWRKY70* gRNA (**Figures 1B–D**). Similarly, the *Nb*WRKY70-YFP signals were also reduced when it was co-expressed with pCas9-*NbWRKY70* gRNA (**Figures 1E–G**). On the contrary, the *Nb*NDR1-YFP fluorescent signals were similar when it was co-expressed with pCas9-*NbEDS1*/*NbNDR1* gRNA, pCas9, or pCas9-*NbWRKY70* gRNA (**Figures 1H–J**). This result again suggests that the gRNA targeting *NbNDR1* is ineffective.

**Figure 2:**
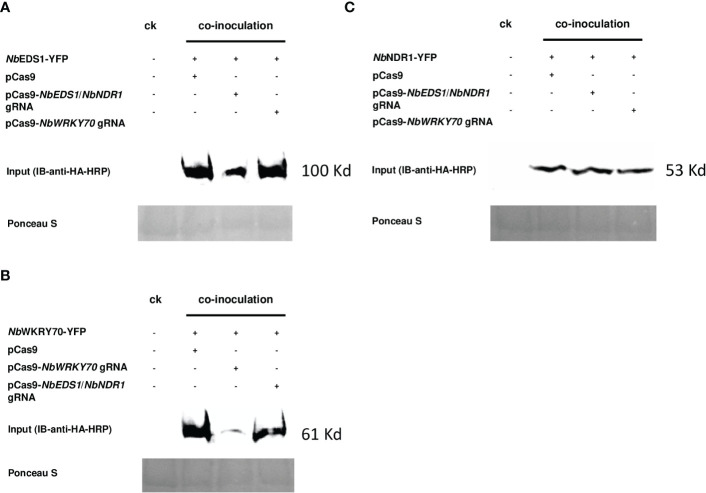
Image of the western blot used to detect the YFP fusion proteins. The protein accumulation of **(A)** *NbEDS1*-YFP, **(B)** *NbWRKY70*-YFP, and **(C)** *NbNDR1*-YFP were detected by probing the blot with anti-HA-HRP antibodies. **(A)** *tumefaciens* strains carrying p35S::*NbEDS1*-YFP, *NbWRKY70*-YFP, and p35S::*NbNDR1*-YFP, were co-inoculated with pCas9 empty vector, pCas9-*NbEDS1*/*NbNDR1* gRNA, and pCas9-*NbWRKY70* gRNA onto the *N. benthamiana* leaf, respectively. CK: the protein was extracted from *N. benthamiana* leaf infiltrated with 10 mM MgCl_2_ that served as a negative control. The blot membrane was stained with Ponceau S to confirm equivalent loadings. +: present of the indicated construct, -: absence of the indicated construct.

Although the reduction in the YFP signal can be detected under a fluorescence microscope, we had never observed that the GFP signals could be suppressed entirely in our transient assays when the GOI-YFP was co-expressed with a cognate Cas9/gRNA. It is not ensured to address a conclusion on gRNA effectiveness based on the reduced YFP signals. To further validate the transient assay result, we performed a western blot analysis to check the accumulation of *Nb*EDS1-YFP, *Nb*NDR1-YFP, and *NbWRKY70*-YFP fusion proteins. The accumulation of *Nb*EDS1-YFP (**Figure 2A**) and *NbWRKY70*-YFP (**Figure 2B**), but not *Nb*NDR1-YFP (**Figure 2C**) fusion proteins, are dramatically reduced when they were co-expressed with the pCas9 construct carrying a corresponding gRNA. This result is consistent with the fluorescent signals detected under a fluorescent microscope (**Figure 1**).

### Development of a pgRNA-YFP vector for functional validation of gRNAs

Although this transient assay method can assess the effectiveness of gRNAs used for CRISPR/Cas9-mediated genome editing in plant cells, it requires the cloning of the open reading frame (ORF) of a target gene, and further validation *via* western blotting analysis. We reasoned that, instead of cloning the ORF of a target gene, we can simply insert the gRNA binding sequence (23 nucleotides including the protospacer adjacent motif (PAM) sequence) into the *YFP* gene, the resulted gRNA-*YFP* fusion gene will have a frameshift in the *YFP* gene, which disrupts the YFP protein expression.

**Figure 3:**
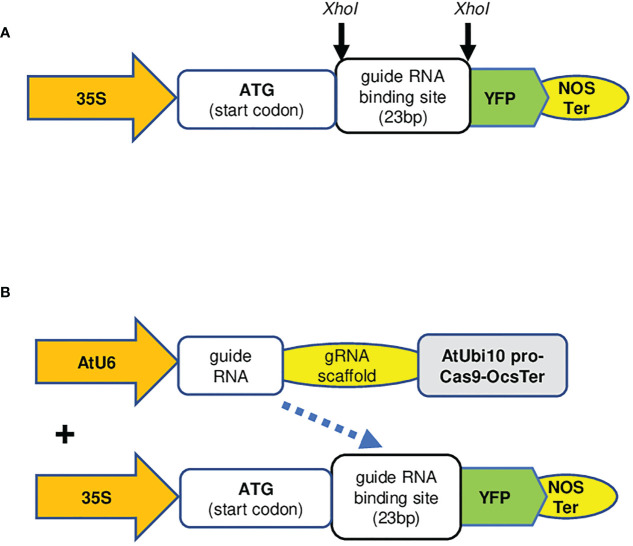
The key elements of the plasmid vectors used in this study. **(A)** The scheme of pgRNA-YFP vector. Two sequence complementary oligonucleotide primers of a given gRNA binding sequence (23 nt), including a PAM site, can be synthesized, annealed, and cloned to the *Xho*I sites of p*Xho*I-ccdB-YFP *via* Gibson cloning. 35S; Cauliflower mosaic virus 35S promoter, YFP; yellow fluorescent protein, Nos Ter; *Agrobacterium tumefaciens* nopaline synthase terminator. Other key elements are identical to the original pEALEYGATE 101 vector [14]. **(B)** The scheme of pCas9-GOI-gRNA and pgRNA-YFP, illustrates that pCas9-AtU6pro-gRNA expressing gRNA targets the *YFP* gene in the pgRNA-YFP construct. AtU6, Arabidopsis U6 promoter; AtUbi10 pro, Arabidopsis ubiquitin 10 promoter; OCS Ter, *Agrobacterium tumefaciens* octopine synthase gene terminator; PAM, protospacer adjacent motif.

To this end, a binary vector, p*Xho*I-YFP, carrying a *YFP* gene, where it has a unique in-frame *Xho*I site adjacent to the start codon of *YFP* gene, was constructed (**Figure 3A**). A synthesized gRNA (23 nt) targeting sequence, including a PAM site, can be inserted into the *Xho*I site causing a frameshift of *YFP* (**Figure 3A**). The derived construct is denominated pgRNA-YFP. When it is co-expressed with another binary vector, pCas9-GOI-gRNA, the *YFP* reading frame could be restored by the indels created *via* CRISPR/Cas9-mediated gene editing events and recover the YFP protein expression (**Figure 3B**). To improve the cloning efficiency, we further modified p*Xho*I-YFP by insertion of an *Xho*I DNA fragment carrying the *ccd*B gene (Liu et al., 2016). The bacterial clones carrying p*Xho*I-ccdB-YFP empty vector can be negatively selected because of the toxicity conditioned by the *ccd*B gene. The derived vector is named p*Xho*I-ccdB-YFP for cloning of any gRNA binding sequences. Cas9 is a large protein with more than 1,300 amino acids, and its expression level in transient assays might be a bottleneck that limits the efficiency of gene editing in a transient assay system. To simplify the protocol and increase gene editing efficiency, we generated a stable transgenic *N. benthamiana* line expressing the *Cas9* gene constitutively (**Figure S3**).

### Functional test of the gRNA screening system *via* Agrobacterium-mediated transient assays

**Figure 4:**
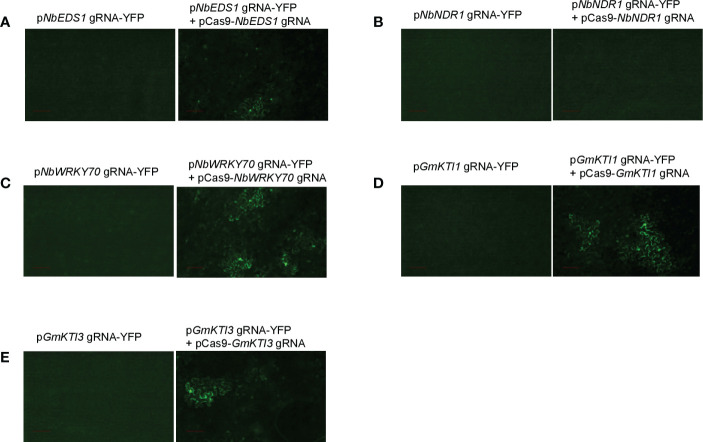
The pgRNA-YFP vectors are used to screen the effectiveness of gRNAs in *Agrobacterium*-mediated transient assays. Transient expression of pgRNA-YFP alone in *N. benthamiana* does not produce any fluorescent signals. **(A)** Co-expression of p*NbEDS1* gRNA-YFP with pCas9-*NbNDR/NbEDS1* gRNA restored the YFP signals in *N. benthamiana*. **(B)** Co-expression of p*NbNDR1* gRNA-YFP with pCas9-*NbNDR1/NbEDS1* gRNA failed to restore the YFP signals. **(C)** Co-expression of p*NbWRKY70* gRNA-YFP with pCas9-*NbWRKY70* gRNA restored the YFP signals. **(D, E)** Co-expression of either p*GmKTI1* gRNA-YFP or p*GmKTI3* gRNA-YFP with pCas9-*GmKTI1/GmKTI3* gRNA restored the YFP signals in *N. benthamiana*. Scale bar represents 100 µm.

We validated the pgRNA-YFP/Cas9 system by re-testing the gRNAs targeting *NbEDS1*, *NbNDR1*, and *NbWRKY70* in *N. benthamiana*. Oligo primers based on the gRNA binding sequence of each gene were synthesized, annealed, and cloned into the *Xho*I site of p*Xho*I-ccdB-YFP. The resulting constructs were p*NbEDS1* gRNA-YFP, p*NbNDR1* gRNA-YFP, and p*NbWRKY70* gRNA-YFP, respectively. The derived gene constructs were co-expressed with pCas9-*NbEDS1*/*NbNDR1* gRNA or pCas9-*Nb*WKRY70 gRNA by *Agrobacterium*-mediated transient assays in wild-type *N. benthamiana* and transgenic *N. benthamiana* harboring the *Cas9* gene. YFP fluorescent signals can be detected when *Cas9* is co-expressed with p*NbEDS1* gRNA-YFP and p*NbWRKY70* gRNA-YFP, but not p*NbNDR1* gRNA-YFP (**Figures 4A–C**). Therefore, we further confirmed that the gRNA targeting *NbNDR1* is ineffective for triggering gene editing, explaining why no *NbNDR1* gene editing events could be detected in stable transgenic plants. These results also imply the feasibility of the new gRNA evaluation system. In addition, we also performed tests on the pCas9 transgenic plants, which works as efficiently as the transient assay method. As previously discussed, the pCas9 transgenic plant makes the system more facile and straightforward. Therefore, we expect the pCas9 transgenic plant could be a valuable resource shared by the plant research community.For the first system described in this study, we have to compare the YFP signals in two samples, where the reduced fluorescent signals could be observed in plant cells with gene editing events. In the second system, the gain of the YFP signals is to compare one sample with YFP signals and another one without fluorescent signals. Therefore, the second method is much more convenient, and the data is more conclusive.

### Evaluate the effectiveness of gRNAs targeting two soybean trypsin inhibitor genes

We further tested the feasibility of predicting the effectiveness of gRNA targeting two soybean genes by using the system described above. To this end, we first validated the gRNA effectiveness and then generated stable transgenic soybean plants. Although successful gene editing events in soybeans have been reported, the low transformation efficiency and a long-tissue culture process impede a broad application of the CRISPR/Cas9 system in soybean molecular breeding programs. Therefore, it is critical to validate the effectiveness of the gRNAs before the initiation of stable soybean transformation experiments.

**Figure 5:**
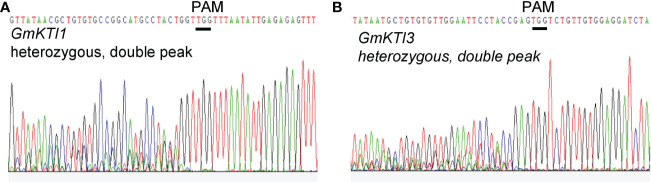
Sanger sequencing chromatograms display the DNA sequence of *GmKTI1* and *GmKTI3* that is targeted for gene editing. The open reading frames of *GmKTI1* and *GmKTI3* in the pCas9-GmKTI1/GmKTI3 gRNA T0 transgenic soybean plants were amplified and sequenced. The double peaks in the sequencing chromatograms indicate the presence of both wild-type and mutant allele of sequences *GmKTI1* **(A)** and *GmKTI3* **(B)**. The wild-type gene sequences are shown on the top of the sequencing chromatograms. The PAM sites are underlined.

Soybean Kunitz trypsin protease inhibitor (*KTI*) genes encode proteins that can reduce protein digestibility in animals and human beings (Gillman et al., 2015). Thus, a KTI-free or low KTI soybean cultivar with mutations on *KTI* genes is highly desirable. We attempted to knock out two seed-specific KTI genes, *KTI1* (Gm01g095000) and *KTI3* (Gm08g341500), *via* CRISPR/Cas9-mediated genome editing approach. The effectiveness of gRNAs targeting *KTI1* and *KTI3* was validated using the transient assay system described above (**Figures 4D, E**). Subsequently, the pCas9-KTI1/3 gRNA construct was transformed into soybean cultivar William 82 *via Agrobacteria*-mediated transformation (Luth et al., 2015). Five independent transgenic plants were recovered and genotyped. Both *KTI1* and *KTI3 gene* editing events were detected in all tested transgenic lines (**Figures 5A, B**).

## Conclusion

In this study, we employed the *Agrobacterium*-mediated transient assays to test the effectiveness of gRNAs. The first method was based on the fact that Cas9 could edit a target gene fused with *YFP* and result in reduced fusion proteins and fluorescent signals in transformed plant cells. The second method, the p*Xho*I-ccdB-YFP/Cas9 system, was developed based on the fact that small indels could be introduced by the nonhomologous end-joining during the process of CRISPR/Cas9-mediated gene editing. Such small indels could correct the reading frame of a *YFP* gene that was disrupted by the insertion of a gRNA binding sequence. We demonstrated that the second method is more convenient and reliable by testing five gRNAs targeting *N. benthamiana* and soybean genes, respectively. The pre-validated gRNAs allow us to generate mutations on target genes in stable transgenic soybean plants (Wang et al., 2022 (under review)). Therefore, we expected the p*Xho*I-ccdB-YFP/Cas9 system to be broadly applicable for screening gRNA(s) for genome editing in crop plants.

## Materials and methods

### Escherichia coli, Agrobacterium tumefaciens and Xanthomonas euvesicatoria strains

*E. coli* strains *DH5a* and *ccdB survivor* (Thermo Fisher Scientific Inc.) were grown on Luria Broth (LB) medium supplemented with appropriate antibiotics at 37°CC*. Agrobacterium tumefaciens strain GV2260* was grown on LB medium at 28°CC. *Xanthomonas euvesicatoria* strain *Xe*85-10 was grown on nutrient yeast glycerol agar (NYGA) medium at 28°CC. Kanamycin 50 ug/ml (Sigma Inc.), Gentamicin 50 ug/ml (Sigma Inc.), and Rifamycin 100 ug/ml (Sigma Inc.) were used to select transformed bacterial cells carrying the designated plasmid constructs.

### Plant materials and growth conditions

Soybean (*Glycine max*, cv. *William 82* (*WM82*)) and *Nicotiana benthamiana* seeds were germinated and grown in Sunshine^®^ Mix #1 in a growth chamber (14hr/10hr light/dark cycle at 25°C/20°C). The 4-week-old plants were used for the experiments detailed herein.

### CRISPR/Cas9 plasmid construction

A previously described pCRISPR/Cas9-Kan construct (Liu et al., 2016) was modified for the application of genome editing in transgenic soybean plants. In brief, we replaced the *Kanamycin* resistance gene cassette with a *Bar* gene cassette (confers resistance to the herbicide Bialaphos). The *Bar* gene cassette which consisted of a mannopine synthase (MAS) promoter, the *Bar* gene open reading frame, and a MAS terminator, was amplified from the plasmid DNA of pEarleyGate101 (Earley et al., 2006). The PCR primers with annotations are listed in **Table S1**. The *Bar* gene cassette was cloned at the *Pme*I/*Mau*BI sites of pCRISPR/Cas9-Kan construct using a Gibson Assembly^®^ Cloning Kit (New England Biolabs Inc). The derived construct was named as pCas9. The Cas9 gene is expressed by using an Arabidopsis ubiquitin 10 gene promoter (Liu et al., 2016). The guiding RNA expression cassettes targeting *GmKTI1* and *GmKTI3*, *NbEDS1* and *NbNDR1*, and *NbWRKY70, respectively*, were synthesized at GenScript Biotech Corp. The expression of gRNA is driven by an Arabidopsis U6 promoter (Liu et al., 2016). The gRNA cassette was cloned into the *Pme*I site of pCas9 using a Gibson Assembly^®^ Cloning Kit. The derived constructs were transformed into *A. tumefaciens* strain *GV2260* for stable transformation and transient gene expression assays.

### Gene cloning and plasmid construction

The open reading frames (ORFs) of *NbEDS1* (Ordon et al., 2017), *NbNDR1* (Dong et al., 2022), and *NbWRKY70* (Liu et al., 2016) were amplified from the cDNA of *N. benthamiana* with primers listed in **Table S1**. The ORFs were cloned into pDonr207^®^ using a BP^®^ cloning kit (Thermo Fisher Scientific). The genes/fragments in donor vectors were subcloned into pEarleyGate101 using an LR^®^ Gateway cloning kit (Thermo Fisher Scientific). The derived constructs are p35S::*NbEDS1*-YFP, p35S::*NbNDR1*-YFP, and p35S::*NbWRKY70*-YFP. The expression of cloned genes is driven by a cauliflower mosaic virus 35S promoter (35S). The plant expression constructs were transformed into *A. tumefaciens* strain *GV2260 via* electroporation.

A binary vector p*Xho*I-YFP carrying a yellow fluorescent protein (*YFP*) gene, was constructed by amplifying and cloning a DNA fragment into the *Xho*I and *Spe*I sites of pEarleyGate101 (Earley et al., 2006). The primers are listed in **Table S1**. The cloned *YFP* gene has a unique in-frame *Xho*I site adjacent to the start codon of *YFP* gene. The ccdB cassette was amplified from pEarleyGate101 and inserted into the *Xho*I site of p*Xho*I-YFP (**Table S1**). The derived vector named p*Xho*I-ccdB-YFP maintained in *E. coli* strain *ccdB* survivor (Thermo Fisher Scientific Inc.), and it can be used for cloning of any synthesized gRNA binding sequences with reduced false-positive clones derived from incomplete digestion of p*Xho*I-ccdB-YFP DNA, which can be negatively selected by the *ccdB* gene in *E. coli DH5α*.

### Gibson cloning

To clone a gRNA binding sequence into p*Xho*I-ccdB-YFP, two sequence complementary oligonucleotide primers of a given gRNA binding sequence (23 nt), including a PAM site, can be synthesized, annealed and cloned to the *Xho*I sites of p*Xho*I-ccdB-YFP *via* Gibson cloning. In brief, two nucleotide primers carrying the gRNA binding sequence, including a PAM site, were synthesized (Integrated DNA Technologies, Inc.). The two primers (100 pm) were annealed by using a thermal cycler programmed as 98 °CC/30 sec, 56 °CC/45 sec, repeated 10 cycles. The annealed gRNA linker was directly cloned into the *Xho*I site of p*Xho*I-ccdB-YFP by using a Gibson cloning kit (New England Inc) (Lei et al., 2014). In brief, 0.01 pmol of linearized vector p*Xho*I-ccdB-YFP, and 0.2 pmol of gRNA linker, were mixed with an equal volume of 2x Gibson master mix, and incubated in a thermal cycler programmed to hold at 50°C for 60 min (Jacobs and Martin, 2016). After incubation, the reaction mixture was transformed into *E. coli DH5a* using a heat shock approach. The derived binary constructs pgRNA-YFP was transformed into *Agrobacterium tumefaciens* strain GV2260 *via* electroporation.

### Agrobacterium-mediated transient assay in N. benthamiana and western blotting analysis

*Agrobacterium* strains *GV2260* harboring pCas9-gRNA plasmid, and pgRNA-YFP plasmid were co-infiltrated into *N. benthamiana* leaf mesophyll tissues for transient protein expression. The YFP signals were monitored using a fluorescent microscope (Axio Observer A.1, Zissis) at 48 hr post infiltration. The YFP gene was fused with a C-terminal HA epitope tag, and the fusion proteins can be detected with anti-HA-HRP (Roche) antibodies at a dilution of 1:5,000. Plant-expressed proteins were detected by Western blot analysis following the procedure as previously described (Wang et al., 2018; Liu et al., 2020b).

### Xanthomonas euvesicatoria disease assay on N. Benthamiana

*Xe* strain *Xe85-10* was cultivated on NYGA mediums supplemented with rifamycin (100 ug/ml) at 28 °CC for two days. The bacterial cells were collected and suspended in 10 mM MgCl_2_ and diluted to 4 x10^8^ CFU ml^-1^. The bacterial inoculum was infiltrated into the backside of *N. benthamiana* leaf using a blunt-end needleless syringe. The inoculated plants were maintained under 14 hr light/10 hr dark at room temperature. The phenotypes of programmed cell death were recorded at 48 hr post-inoculation.

### Generation of transgenic tobacco and soybean plant by Agrobacterium

*Agrobacterium* strain *GV2260* harboring plasmid pCas9, or pCas9-gRNA was used for tobacco transformation following a protocol as previously described (Liu et al., 2016). Soybean transformation was performed at the plant transformation facility at Iowa State University as previously described (Paz et al., 2006; Luth et al., 2015; Ge et al., 2016).

## Data availability statement

The original contributions presented in the study are included in the article/**Supplementary materials**. Further inquiries can be directed to the corresponding authors.

## Author contributions

BZhao conceived the project; ZW and BZhao designed the research procedure. ZW, ZS, KW, QL, KM, performed the experiments. ZW and BZhao analyzed the data. ZW, BZhang, and BZhao wrote the manuscript with input from all authors. All authors contributed to the article and approved the submitted version.

## Supplementary material

Supplementary Figure 2 Knock out of *NbEDS1* impairs the cell death trigerd by interaction of *Nb*Roq1 and XopQ, but not Rps2/AvrRpt2. **(A)** The CRISPR/Cas9 construct harbors three necessary elements exhibited as below: the plant selection cassette consists of a MAS (mannopine synthase) promoter, Bar gene, and a MAS terminator; the Cas9 cassette consists of *Arabidopsis* ubiquitin 10 promoter, Cas9 gene, and OCS (octopine synthase) terminator; two guide RNA cassettes and each of them consists of a *Arabidopsis* U6 promoter, and one sgRNA. Sequence alignment of the wild type and mutant *EDS1* **(B)** or *NDR1* **(C)** alleles generated *via* CRISPR/Cas9-mediated genome editing displayed that the *NbEDS1* gene was successfully edited in all genotyped plants, while no edited *NbNDR1* genes were detected in any genotyped plants. **(D)** The pCas9- *NbNDR1*/*NbEDS1* gRNA transgenic plants and wild-type plants were inoculated with *Xanthomonas euvesicatoria* strain Xe85-10. By contrast, the co-inoculation of *A. tumefaciens* strains carrying p35S::*RPS2* and p35S::*avrRpt2*, both with the concentration of 2 x10^8^ CFU ml^-1^, can trigger cell death on both wild-type and transgenic *N. benthamiana* at 48 hpi **(E)**. The pictures were taken from the front side and back side of the leaf sample.

Supplementary Figure 3 Validation of the pCas9 transgenic *N. benthamiana* plants. The pCas9 vector that doesn’t carry any gRNAs was used to transform *N. benthamiana* plant. The *Cas9* gene fragments were detected in transgenic lines #1 and #2 by PCR amplification but not in wild- type plants. The plasmid DNA pCas9 is served as the template for positive control. (1) 1 Kb marker, (2) pCas9 line 1, (3) pCas9 line 2, (4) wild-type plant, (5) pCas9 plasmid DNA.
