# Supplementary material for: Evaluate the guide RNA effectiveness via Agrobacterium-mediated transient assays in Nicotiana benthamiana
Source: Front Plant Sci. 2023 Feb 20;14:1111683. doi: 10.3389/fpls.2023.1111683 (PMC9986745; doi:10.3389/fpls.2023.1111683)
Supplement: Supplementary Figure 2 — Knock out of NbEDS1 impairs the cell death trigerd by interaction of NbRoq1 and XopQ, but not Rps2/AvrRpt2. (A) The CRISPR/Cas9 construct harbors three necessary elements exhibited as below: the plant selection cassette consists of a MAS (mannopine synthase) promoter, Bar gene, and a MAS terminator; the Cas9 cassette consists of Arabidopsis ubiquitin 10 promoter, Cas9 gene, and OCS (octopine synthase) terminator; two guide RNA cassettes and each of them consists of a Arabidopsis U6 promoter, and one sgRNA. Sequence alignment of the wild type and mutant EDS1 (B) or NDR1 (C) alleles generated via CRISPR/Cas9-mediated genome editing displayed that the NbEDS1 gene was successfully edited in all genotyped plants, while no edited NbNDR1 genes were detected in any genotyped plants. (D) The pCas9- NbNDR1/NbEDS1 gRNA transgenic plants and wild-type plants were inoculated with Xanthomonas euvesicatoria strain Xe85-10. By contrast, the co-inoculation of A. tumefaciens strains carrying p35S::RPS2 and p35S::avrRpt2, both with the concentration of 2 x10^8^ CFU ml-1, can trigger cell death on both wild-type and transgenic N. benthamiana at 48 hpi (E). The pictures were taken from the front side and back side of the leaf sample. [file Image_1.pdf]

Figure S1. The nucleotide sequences of *NbEDS1*a, *NbEDS1*b, and *NbNDR1*. The guide RNA binding sequence is highlighted in yellow; the PAM sites are highlighted in blue. The start and stop codons of *NbEDS1* and *NbNDR1*genes are in red.

NbDES1a 1 **ATG**GTGAGAATTGAAGAGGGGAGAGAAGTGAAAGATGAGCTGATCAAGAAAGCTTGTAACTTAGCAATGGAAGCTCACAGTTTGTCTTCTGGGAAGCCTTATATTTTCAAGAAAAAAAGT

NbEDS1b 1 **ATG**GTGAGAATTGAAGAGGGGAGAGAAGTGAAAGATGAGCTGATCAAGAAAGCTTGTAACTTAGCAATGGAAGCTCACAGTTTGTCTTCTGGGAAGCCTTATATTTTCAAGAAAAAAAGT

NbDES1a 121 GGATCGATGGATGTTTTTTTGGCTTTTGCTGGAAATTGGTCTGTTGATGGTTGGTACAGTTGTAGCACTTCTTTTGGAGAGAAAAAATCTTCGTTTCCATCATTGAAAAGTGTTGGCACA

NbEDS1b 121 GGATCGATGGATGTTTTTTTGGCTTTTGCTGGAAATTGGTCTGTTGATGGTTGGTACAGTTGTAGCACTTCTTTTGGAGAGAAAAAATCTTCGTTTCCATCATTGAAAAGTGTTGGCACA

NbDES1a 241 GATGAGGTAGCCATGGTTAATGAAGCATTTGCTAGCAGATTTGAACACATATTGAACAACTCTTCTCTTAAAAATGAGGTACTGTGTTTGATAATCTTGTTCCTTTTCTTTCTCTTTTTT

NbEDS1b 241 GATGAGGTAGCCATGGTTAATGAAGCATTTGCTAGCAGATTTGAACACATATTGAACAACTCTTCTCTTAAAAATGAGGTACTGTGTTTGATAATCTTGTTCCTTTTCTTTCTCTTTTTT

NbDES1a 361 GGGTGGTATTTTGTTTTTCGTTTTGGGTGATTAAATGTTTAACTTCCGTGCACTGACAATGTACAGTAGCAAATTAAGGTGACTTGAACAACAGTGTTATAACAATTTAGACTATAGTGA

NbEDS1b 361 GGGTGGTATTTTGTTTTTCGTTTTGGGTGATTAAATGTTTAACTTCCGTGCACTGACAATGTACAGTAGCAAATTAAGGTGACTTGAACAACAGTGTTATAACAATTTAGACTATAGTGA

NbDES1a 481 CAATGTTAGAATTCTGTACACTGTCTTAAATATGATAATTAGTTACCTAAATAGGAAGTGAATTGTGGAAAAAGATTCAATTTTTTCATTTTTGAGGTGGGGTTTTGAAGGGGGGACAGT

NbEDS1b 481 CAATGTTAGAATTCTGTACACTGTCTTAAATATGATAATTAGTTACCTAAATAGGAAGTGAATTGTGGAAAAAGATTCAATTTTTTCATTTTTGAGGTGGGGTTTTGAAGGGGGGACAGT

NbDES1a 601 TCCAGAATGGATTAGCACATAGATTGAATATTGCCTCGTTCTTTTTAGACAAAGGTTTTCACAGCTGAGTGTAAACCTGTCCCCCAAAAAAATCTTCTTTGGTGATCTAGAACTGATGTG

NbEDS1b 601 TCCAGAATGGATTAGCACATAGATTGAATATTGCCTCGTTCTTTTTAGACAAAGGTTTTCACAGCTGAGTGTAAACCTGTCCCCCAAAAAAATCTTCTTTGGTGATCTAGAACTGATGTG

NbDES1a 721 AGTATTAGTTTTATTGCCTCGTCTCCGGGCAGAGACGATGCAATCTTTGTGTAACGGGTTCAACTAAACTCAATATTTTTGACACATATATATATTTAGAAGTACAATGAATTCAGTCTA

NbEDS1b 721 AGTATTAGTTTTATTGCCTCGTCTCCGGGCAGAGACGATGCAATCTTTGTGTAACGGGTTCAACTAAACTCAATATTTTTGACACATATATATATTTAGAAGTACAATGAATTCAGTCTA

NbDES1a 841 AAAACCTTAAAGGTTAAATCAATCATGTTTAAATCCTAAATCCTCCTCTCCCCCAGGACCAGCTTTGTCAATTTCAACAATGGAAGACAGAGAGGGTTATACTCAGCGTCAAGAAGTTCC

NbEDS1b 841 AAAACCTTAAAGGTTAAATCAATCATGTTTAAATCCTAAATCCTCCTCTCCCCCAGGACCAGCTTTGTCAATTTCAACAATGGAAGACAGAGAGGGTTATACTCAGCGTCAAGAAGTTCC

NbDES1a 961 ACTCCTGACAATATCATGCTGATCTAGCTAACTTAATTCCTCTTTATTAGCTCTTAGTTATCAAATCTTCATGTCCAAGAATATTTTTCCAAGTCAAACTAAAACACCGATTCCTATCAA

NbEDS1b 961 ACTCCTGACAATATCATGCTGATCTAGCTAACTTAATTCCTCTTTATTAGCTCTTAGTTATCAAATCTTCATGTCCAAGAATATTTTTCCAAGTCAAACTAAAACACCGATTCCTATCAA

NbDES1a 1081 ATATTCTTCAAGATGATTCCTCTTTAGACTAACAAAGATTAACCTTCATAGCTTCATCTTTAATTTGGAAACTGAATGTTGATTCATGGGAACTGTTCGTTATAGGTGGAGAAGGCGATG

NbEDS1b 1081 ATATTCTTCAAGATGATTCCTCTTTAGACTAACAAAGATTAACCTTCATAGCTTCATCTTTAATTTGGAAACTGAATGTTGATTCATGGGAACTGTTCGTTATAGGTGGAGAAGGCGATG

NbDES1a 1201 TCAGAAGGAAAACAGATAGTGTTTGCAGGGCACTCGTCGGGTGGCCCTATTGCGATTTTGGCAGCCCTATGGTGTCTAGAACATTGTTGCACAAGACCAAATGACAACCTGGTTTGTCCA

NbEDS1b 1201 TCAGAAGGAAAACAGATAGTGTTTGCAGGGCACTCGTCGGGTGGCCCTATTGCGATTTTGGCAGCCCTATGGTGTCTAGAACATTGTTGCACAAGACCAAATGACAACCTGGTTTGTCCA

NbDES1a 1321 TACTGTATAACTTTTGGATGCCCTCTTGTTGGTGACAGAATATGGTCTCATGCCCTCATGCGCGAAAACTGGGCTCGTTACTTCATACATTTTGTCACGAAATATGATATCGTTCCTCGG

NbEDS1b 1321 TACTGTATAACTTTTGGATGCCCTCTTGTTGGTGACAGAATATGGTCTCATGCCCTCATGCGCGAAAACTGGGCTCGTTACTTCATACATTTTGTCACGAAATATGATATCGTTCCTCGG

NbDES1a 1441 ATGATGCTTGCTCCCCTTTCATCGATTCAAGAATGGCTTCAAGCAATCTTTGACTTCATCAATCCAAAATCCCGGAATTATCAGCACGAGGTAGTTGTAAGATCATATGATGCATCGAAG

NbEDS1b 1441 ATGATGCTTGCTCCCCTTTCATCGATTCAAGAATGGCTTCAAGCAATCTTTGACTTCATCAATCCAAAATCCCGGAATTATCAGCACGAGGTAGTTGTAAGATCATATGATGCATCGAAG

NbDES1a 1561 AATTTCTTTATGACTGTAATGAGGAGTGCATCCTCTGTTGCAAGTTATGCTGCATGTAATCTGAAAGGATGCACAAACTTGTTGTTAGAAACAGTTTCTAACATTGTTCAACTCAGCCCT

NbEDS1b 1561 AATTTCTTTATGACTGTAATGAGGAGTGCATCCTCTGTTGCAAGTTATGCTGCATGTAATCTGAAAGGATGCACAAACTTGTTGTTAGAAACAGTTTCTAACATTGTTCAACTCAGCCCT

NbDES1a 1681 TATAGACCTTTTGGAACTTACATCTTCTGCACTGGAAATGGGAAACTGGTGGTCGTTGAGAATCCAGATGCTGTTCTGCAGTTACTGTTCTATTGTGCTCAAATGAGTTCCGAAACAGAA

NbEDS1b 1681 TATAGACCTTTTGGAACTTACATCTTCTGCACTGGAAATGGGAAACTGGTGGTCGTTGAGAATCCAGATGCTGTTCTGCAGTTACTGTTCTATTGTGCTCAAATGAGTTCCGAAACAGAA

NbDES1a 1801 GTTGAAGAAGTTGTTACCAGAAGCTTAAACGAACATTTGTTATATAGAAAAGAAATGCAGGAAAGCTTAGAGATGCAGGATGTGGTTCATCTCAATAATCTTACCGATATTCCCTTGTCT

NbEDS1b 1801 GTTGAAGAAGTTGTTACCAGAAGCTTAAACGAACATTTGTTATATAGAAAAGAAATGCAGGAAAGCTTAGAGATGCAGGATGTGGTTCATCTCAATAATCTTACCGATATTCCCTTGTCT

NbDES1a 1921 TCAAATGCCATTGCATTAGCTAGTGATGAAGTGGTAACGATGAATTTAGCCCTGAATGACTTAGGCCTGGTAAGTGATAAATGTGTTTGCTCTTTGTGCTGATTTTCACATTTAGCTCAA

NbEDS1b 1921 TCAAATGCCATTGCATTAGCTAGTGATGAAGTGGTAACGATGAATTTAGCCCTGAATGACTTAGGCCTGGTAAGTGATAAATGTGTTTGCTCTTTGTGCTGATTTTCACATTTAGCTCAA

NbDES1a 2041 AGTAACGGGTTTCTTTTTCACGTTACAGAGTACAAGAGCACGGTTGTGTCTTCGTGCAGCAGGACAATGGGAGAAGCAGAAAAGGAAGAACGAGGAAAAGATTGATGGTAATAAGAACAG

NbEDS1b 2041 AGTAACGGGTTTCTTTTTCACGTTACAGAGTACAAGAGCACGGTTGTGTCTTCGTGCAGCAGGACAATGGGAGAAGCAGAAAAGGAAGAACGAGGAAAAGATTGATGGTAATAAGAACAG

NbDES1a 2161 CATCATGGAAGGATTAAGCAAGATACAGGAGTACCAGACCAAGTGTGATATTCAGAAAGTCGGGTATTATGATGCGTTCAAGCTTCAAGAGACCATCGATGACTTCAATGCTAATGTGAA

NbEDS1b 2161 CATCATGGAAGGATTAAGCAAGATACAGGAGTACCAGACCAAGTGTGATATTCAGAAAGTCGGGTATTATGATGCGTTCAAGCTTCAAGAGACCATCGATGACTTCAATGCTAATGTGAA

NbDES1a 2281 AAGGCTGGAGCTAGCAGGAATATGGGACGAAATCATTGAAATGTTGAAAAGGTATGAGCTCCCAGATAGTTTTGAGGGAAGAAAGGAATGGATAAAACTAGGGACGCAGTTCCGCAGGCA

NbEDS1b 2281 AAGGCTGGAGCTAGCAGGAATATGGGACGAAATCATTGAAATGTTGAAAAGGTATGAGCTCCCAGATAGTTTTGAGGGAAGAAAGGAATGGATAAAACTAGGGACGCAGTTCCGCAGGCA

NbDES1a 2401 AGTTGAGCCCTTGGATATTGCAAACTATTACAGGCATTTGAAGAATGAAGATACAGGACCTTACATGATCAGGGCTAGGCCGAAGCGTTATAGGTTCACACAACGATGGTTAGAGCATGA

NbEDS1b 2401 AGTTGAGCCCTTGGATATTGCAAACTATTACAGGCATTTGAAGAATGAAGATACAGGACCTTACATGATCAGGGCTAGGCCGAAGCGTTATAGGTTCACACAACGATGGTTAGAGCATGA

NbDES1a 2521 AGAGAGGGTGCAAACAGGTGAACGCTCTGAGTCTTGTTTTTGGGCAGAAGTGGAGGAACTAAGAAACAAGCCAATTATGGAAGTGCAAAACAGGATTTTGAGTTTAGAAACAAAGGCATG

NbEDS1b 2521 AGAGAGGGTGCAAACAGGTGAACGCTCTGAGTCTTGTTTTTGGGCAGAAGTGGAGGAACTAAGAAACAAGCCAATTATGGAAGTGCAAAACAGGATTTTGAGTTTAGAAACAAAGGCATG

NbDES1a 2641 GGATTGGTCCCAGAGTGGCCTTCTGGGCGATGATGTTTTCTTCCCTGAGTCTACCTTTACCAAATGGTGGAAACAACTCCCTACTCAGCACAGAATGACATCTTGGATATCAGGGAAAGT

NbEDS1b 2641 GGATTGGTCCCAGAGTGGCCTTCTGGGCGATGATGTTTTCTTCCCTGAGTCTACCTTTACCAAATGGTGGAAACAACTCCCTACTCAGCACAGAATGACATCTTGGATATCAGGGAAAGT

NbDES1a 2761 AAATTCT**TAG**

NbEDS1b 2761 AAATTCT**TAG**

> *Nicotiana benthamiana* cDNA sequence of *NbNDR1*

TGAAAATAGACTAACCATAACCTTGAGTAAGAAATGAATATTTTTGACAAAAAATAACACGTCTAATTACATAGATAGCGTAAAAGAATTTACACTATCAAATAAATACACTCCCTCTATTCTA

TTTTTTAAGGCATTATTTCTTTTATTTGTCTATTCCCAAAAAAATCCAGTTTCTATTTTTAAAACTAATAAAATTACGAAATTCTCACTTTATCCTAAATGATAAATTTTTATAATTATATAAA

TATTTTTTCCTTTTTAACCCCTAACACACACAAACCGGCCACCTAGACTCCTTTTTTATTGGCTAGTTTGCAGCTTGACCCCTTGTCATTGACTTGAAGGTCACCAAGATTTTAATTGGCGCTC

ACTTACCCAATTACCAGAAATTCATAGGGCTGCTGCTGCTGCTTACATACTCATTTACTAGCACTTCCTCAAAGCAGAAAGAAAAACAAAAAGCAAAAAGGACAGGGACAAAAGTATAATTGCC

CTATAGATCTTTTTATATAAACCCAAGATTTTCTTTTTCTCAAACAACATATTCTTCAAGTTTCAATTTTGATCTACCCTTTCATATATTTTCCTATTTTTCAGATTCTTGAAAAACCAATTTT

TTTCAAC**ATG**TCAGACTATGGATCCAATAATACATGCTGCTTTAAGTGCATCCAATTCATATTAACAGCAGGCTTAACAGCTCTTTTCATTTGGCTAAGTCTAAGAACCACAAAACCATCTTGC

TCTTTAGATAATTTTTACTTACCTGCCCTTAACAACTCTGATAACTCCAACTCCACAAGATCCAATCATACACTTTCCTTTCAGCTCAATTTGAACAACAAGATGAAGGACAAAGGTGTTCGTT

ACGATGACATTAAACTTAGTTTTTATTATGGTACAAATACAAGTTTTCCTATAGGTAATTATACAGTACCTGGCTTTTACCAAGGTCATGACAAGAAAGCACATAAAAAAGGTATACTGGAAAC

TCAGAAAATGCCTTGGAATGATGCTTTAAAGATGGTTTCAAATGGGTCTAAAGTGGTTTTCAGGGTGGATGTAGCTACTAGAGTCAGGTACAAGGTCATTCTTTGGTATACTAAGAGGCATAAT

TTTACTGTGGAAAATAGTAAAGTGAAAGTGGATGGTTCAGGTAAATCAAGCGCACAGAAGCTTCATTGCTGTTTTATGACTTTTGGACTTCCCCGAATTGTTTTATTCACCTTGCTTCTTTTGT

TG**TAA**AAGATTCCTCGGGTATTCAAGATTTAAGCTTGATTAGTTCAGTTTTACAATTGCAGGTTCAGGTTTTAGTATTTGGTAACATTCTAGTAATTCTTTTAATTATGTATCATTTAAAAAAT

GTTGGACTCAGTTGATTGCATGCTGTCGGTGCAAACTGACTGTAGTTGGTGTAATCTTTTCTTTTTGCATTTATTCTTTATAAATTTTTACTGGCTTGTCTTATGTGGTAGATCCTATGATCTT

TTTTCTTGGATATCATTGTTTTTACTCATTCTATTTACTAGGTTAATATTATTCATTTCATATTGATAAGTGGCTATGAAAGAAGCGTGTTCCTATATTATGTTGTATTATTGATGTCACAGGC

CTTATCTTTTCCTGTCTTCCCACCTTTCTCTTTTTAATGAGCCATATGGACTTTGGTCCACATTAATTAAAGGGATAGTTTATGATTCTTTGCTTTCTGATGCCTTCTCAGGAGTATGATTTGT

AGAATAATGCCCTCCAGATATATTCTATCCAACTAGGGTTGTCCTAATTATTATTCCTATATAAAAAAAAAAAGTGAAAAGCATAGAATATAATTCTTCGGGAATGGGTGTTCTTCTGATTTAGT

ATTTTGAATAGCAACTTCAACAAAAAATTATATAATAATTAAATAGACTTGATGGATAGGGAATAAAATCTGATTCCCTTTTAAGGATTTAAATTAGAATTTTGGTTTTATCCTGAATCCTGAT

AAAAAGAACTTGTAAAAT

Figure S2

A

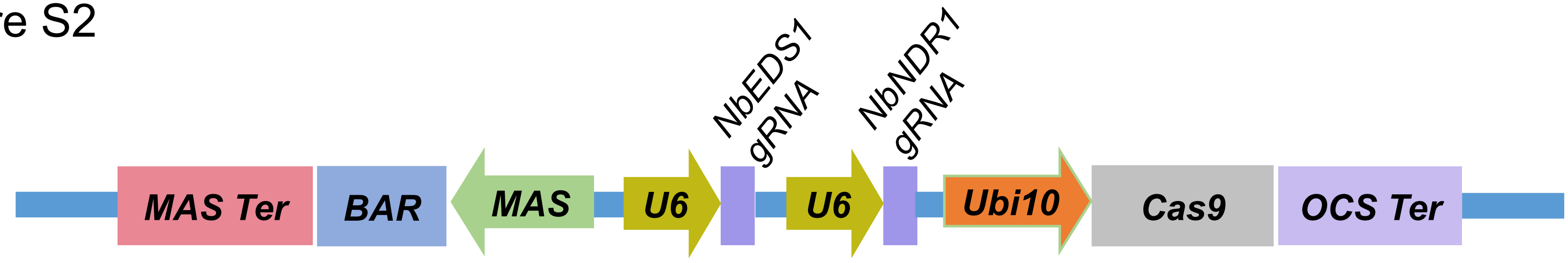

B

```
wt    TTTTGCTGGAAATTGGTCTGTTGATG-GTTGGTACAGTTGTAGCACTTCTTTTGGAG
#15   TTTTGCTGGAAATTGGTCTGTTGATTGGTTGGTACAGTTGTAGCACTTCTTTTGGAG
#19   TTTTGCTGGAAATTGGTTTGTTG----ATTGGTACAGTTGTAGCACTTCTTTTGGAG
```

C

```
wt    TTTAAGTGCATCCAATTCATATTAACAGCAGGCTTAACAGCTCTTTTCATTTGGCTA
#4    TTTAAGTGCATCCAATTCATATTAACAGCAGGCTTAACAGCTCTTTTCATTTGGCTA
#7    TTTAAGTGCATCCAATTCATATTAACAGCAGGCTTAACAGCTCTTTTCATTTGGCTA
#12   TTTAAGTGCATCCAATTCATATTAACAGCAGGCTTAACAGCTCTTTTCATTTGGCTA
#13   TTTAAGTGCATCCAATTCATATTAACAGCAGGCTTAACAGCTCTTTTCATTTGGCTA
#15   TTTAAGTGCATCCAATTCATATTAACAGCAGGCTTAACAGCTCTTTTCATTTGGCTA
#19   TTTAAGTGCATCCAATTCATATTAACAGCAGGCTTAACAGCTCTTTTCATTTGGCTA
```

D **E**

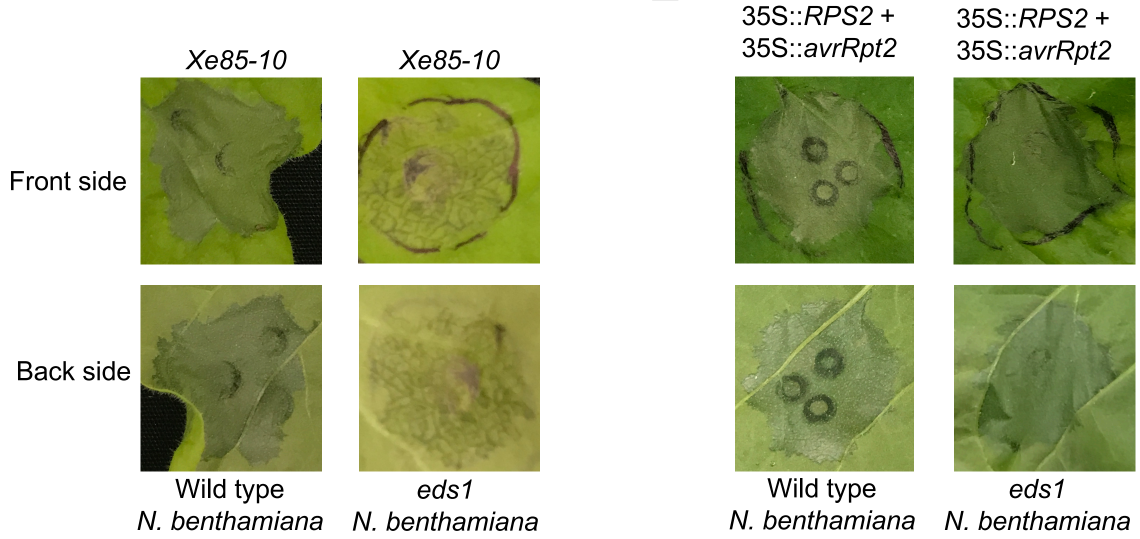

Figure S3

1 2 3 4 5

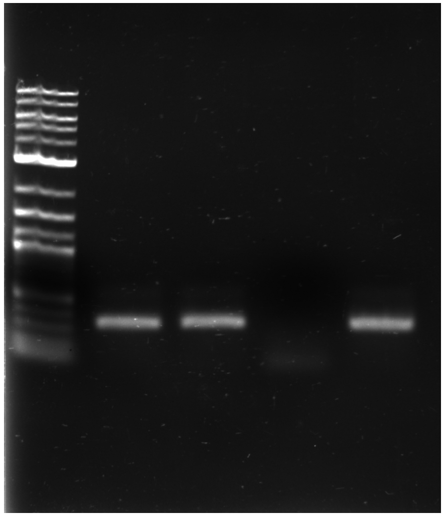

371 bp, Cas9 fragment
