# Supplementary material for: Evaluate the guide RNA effectiveness via Agrobacterium-mediated transient assays in Nicotiana benthamiana
Source: Front Plant Sci. 2023 Feb 20;14:1111683. doi: 10.3389/fpls.2023.1111683 (PMC9986745; doi:10.3389/fpls.2023.1111683)
Supplement: Supplementary file 2 [file Table_1.docx]

**Table S1.** Oligo primers used in this study.

| **Primer** | **Sequence** | **Annotation** |
| --- | --- | --- |
| *NbEDS1* For | GGGGACAAGTTTGTACAAAAAAGCAGGCTtggaattcATGGTGAGAATTGAAGAGGG | Forward primer for amplifying *NbEDS1* ORF |
| *NbEDS1* Rev | GGGGACCACTTTGTAcaAGAAAGCTGGGTactcgagAGAATTTACTTTCCCTGATATC | Reverse primer for amplifying *NbEDS1* ORF |
| *NbNDR1* For | GGGGACAAGTTTGTACAAAAAAGCAGGCTtggaattcATGTCAGACTATGGATCCAAT | Forward primer for amplifying *NbNDR1* ORF |
| *NbNDR1* Rev | GGGGACCACTTTGTAcaAGAAAGCTGGGTactcgagCAACAAAAGAAGCAAGGTGAATAAAACA | Reverse primer for amplifying *NbNDR1* ORF |
| *NbWRKY70* For | GGGGACAAGTTTGTACAAAAAAGCAGGCTtgATGGAGTCTCCGTTGCCGGAAAAG | Forward primer for amplifying *NbWRKY70* ORF |
| *NbWRKY70* Rev | GGGGACCACTTTGTAcaAGAAAGCTGGGTaAGAATTGTACCCTTCAAAAT | Reverse primer for amplifying *NbWRKY70* ORF |
| MAS pro For | ATCCGTAGCATACTAGCATCTATCAGCTAGCgtttaaacCGGCTACCGATCGACTGACTAGCATGATGATaaacTTTTCAAATCAGTGCGCAAGACG | Forward primer for amplifying the cassette composed of MAS promoter, BAR gene, and MAS terminator, and clone to the *Pme*I/*Mau*BI sites of pCas9-Kan vector (Liu et al., 2016) |
| MAS ter Rev | cgatctagtaacatagatgacaccgcgcgcgGATAATTTATTTGAAAATTCATAAGA | Reverse primer for amplifying the cassette composed of MAS promoter, BAR gene, and MAS terminator, and clone to the *Pme*I/*Mau*BI sites of pCas9-Kan vector (Liu et al., 2016) |
| YFP *Xho*I For | CATTTCATTTGGAGAGGACACGctcgaAGTCGCCACCatgAGCAAGGGCGAGGAGctcgagCTGTTCACCGGGGTGGT | Forward primer for amplifying the YFP gene and cloned it into the *Xho*I site of pEarleyGate101 |
| YFP *Xho*I Rev | GCGGTACCGTCGACTGCAGAATTCGAAGCTTGAGCTCGActTCTGAGTCCGGACTTG | Reverse primer for amplifying the YFP gene and cloned it into the *Xho*I site of pEarleyGate101 |
| guide*Xho*I-CmccdB-*Xho* for, | tcgaAatgAGCAAGGGCGAGGAGctcgagGCGGCCGCATTAGGCACCCCAGGCTTTACAC | Forward primer for amplifying *the ccdB* cassette and clone it to the *Xho*I site of p*Xho*I-YFP. |
| guide*Xho*I-CmccdB-*Xho* rev, | ATGGGCACCACCCCGGTGAACAGctcgagTTATATTCCCCAGAACATCA | Forward primer for amplifying the *ccdB* cassette and clone it to the *Xho*I site of p*Xho*I-YFP. |
| *Xho*I *NbNDR1* gRNA for | tcgaAatgAGCAAGGGCGAGGAGctcgagATCCAATTCATATTAACAGC | Forward primer carrying the *NbNDR1* gRNA binding sequence |
| *Xho*I *NbNDR1* gRNA rev | ATGGGCACCACCCCGGTGAACAGctcgagcctGCTGTTAATATGAATTGGAT | Reverse primer carrying the *NbNDR1* gRNA binding sequence |
| *Xho*I *NbEDS1* gRNA for | tcgaAatgAGCAAGGGCGAGGAGctcgagGAAATTGGTCTGTTGATGGT | Forward primer carrying the *NbEDS1* gRNA binding sequence |
| *Xho*I *NbEDS1* gRNA rev | ATGGGCACCACCCCGGTGAACAGctcgagccaACCATCAACAGACCAATTTC | Reverse primer carrying the *NbEDS1* gRNA binding sequence |
| *Xho*I *NbWRKY70* gRNA for | tcgaAatgAGCAAGGGCGAGGAGctcgagGCAATCGACGGGTTAATTCG | Forward primer carrying the *NbWRKY70* gRNA binding sequence |
| *Xho*I *NbWRKY70* gRNA rev | ATGGGCACCACCCCGGTGAACAGctcgagccaCGAATTAACCCGTCGATTGC | Reverse primer carrying the *NbWRKY70* gRNA binding sequence |
| *Xho*I GmKTI1 gRNA for | tcgaAatgAGCAAGGGCGAGGAGctcgagCACGCGATACAGTAGATGGT | Forward primer carrying the GmKTI1 gRNA binding sequence |
| *Xho*I GmKTI1 gRNA rev | ATGGGCACCACCCCGGTGAACAGctcgagccaACCATCTACTGTATCGCGTG | Reverse primer carrying the GmKTI1 gRNA binding sequence |
| *Xho*I GmKTI3 gRNA for | tcgaAatgAGCAAGGGCGAGGAGctcgagGTGTTGGAATTCCTACCGAG | Forward primer carrying the GmKTI3 gRNA binding sequence |
| *Xho*I GmKTI3 gRNA rev | ATGGGCACCACCCCGGTGAACAGctcgagccaCTCGGTAGGAATTCCAACAC | Reverse primer carrying the GmKTI3 gRNA binding sequence |
